# Purpose in Life and Positive Health Outcomes Among Older Adults

**DOI:** 10.1089/pop.2017.0063

**Published:** 2018-04-01

**Authors:** Shirley Musich, Shaohung S. Wang, Sandra Kraemer, Kevin Hawkins, Ellen Wicker

**Affiliations:** ^1^ Advanced Analytics, Optum, Ann Arbor, Michigan.; ^2^ Medicare and Retirement, UnitedHealthcare Alliances, Minneapolis, Minnesota.; ^3^ AARP Services, Inc., Washington, District of Columbia.

**Keywords:** purpose in life, health outcomes, older adults, Medicare, Medicare Supplement

## Abstract

Purpose in life (PIL) is conceptualized as having goals, a sense of direction, and a feeling that there is meaning to present and past life. PIL has been associated with positive health outcomes among older adults, including fewer chronic conditions, less disability, and reduced mortality. The purpose of this study was to estimate the prevalence of PIL among AARP Medicare Supplement insureds, identify associated characteristics, and measure impact on selected health outcomes. In 2016, surveys were sent to a random stratified sample; PIL was measured using a 7-item scale with 5 responses. Scores were averaged across responses and categorized to PIL levels of low, medium, and high. Survey responses were weighted to adjust for nonresponse bias and to weight to a nationally representative population. Multivariate regression models, adjusting for confounding covariates, were utilized to determine characteristics associated with PIL levels and the impact on health care utilization and expenditures, preventive services compliance and quality of life (QOL). Among weighted survey respondents (N = 15,680), low, medium, and high PIL levels were 24.2%, 21.1%, and 54.7%, respectively. The strongest characteristics of medium and high PIL included social support, resilience, reliance on faith, high health literacy, and good health status. Individuals with medium and high PIL had significantly lower health care utilization and expenditures, increased preventive services compliance, and higher QOL. PIL is strongly associated with improved mental and physical health outcomes among older adults. Thus, interventions to improve and/or maintain higher levels of PIL over time may promote successful aging.

## Introduction

As populations age, successful aging is a concept that has gained increasing importance.^1–4^ Current generations of older adults expect to age well, maintaining their mental and physical well-being and enhancing the quality of their later years.^3^ Although the necessary drivers of successful aging are not completely understood, the qualities of aging well in a population are recognizable. Certainly, successful aging is more than longevity or the absence of disease and disability; rather successful aging implies health, physical functionality, and psychological well-being.^1–4^ However, most health-related research and the majority of designed interventions have targeted the negative aspects of health: chronic disease, health risks, physical disabilities, and negative psychological states (eg, depression and anxiety).^5–11^ Research on the positive determinants of health and strategies for keeping healthy people healthy over time has received less attention.^5^

One of the earliest aging models, the compression of morbidity theory,^12^ proposed a lifestyle approach to aging well. Subsequently, long-term prospective studies have documented the benefits of physical activity and healthy lifestyle behaviors in midlife as strong predictors for delays in the onset of disease and disability in later life.^12,13^ As successful aging models have developed, the impact of psychological well-being on physical health has been explored in greater depth.^1–4^ These models have incorporated concepts such as resilience, social support, and purpose in life (PIL) as contributors to positive health over time. Meanwhile, research on the physical mechanisms explaining the impact of psychological well-being on physical health has focused largely on the actions of buffering the negative impacts of stress on health.^4,14–17^ Physical biomarker evidence is consistent with this hypothesis, indicating those with high PIL or strong social support demonstrate lower cardiovascular, endocrine, and immune biochemical markers.^18^

Of primary interest in this study, PIL has various definitions but generally is conceptualized as having goals, a sense of direction, and a feeling that there is meaning to present and past life.^6–9,19–22^ In numerous prospective research studies, higher PIL has been associated with better self-rated health,^22^ fewer chronic diseases (eg, stroke,^8,19^ myocardial infarctions^9^), reduced pain,^11^ less disability,^7,10,11^ less dementia and Alzheimer's disease,^23^ and reduced mortality.^6,10,11^ In addition, those with higher PIL are more compliant with preventive services,^20^ are more likely to be physically active,^11,21,24^ engaged in meaningful activities,^24^ and have fewer sleep problems.^17^ As currently measured on a number of different scales, PIL as a construct is stable over long periods of time, decreasing slowly from midlife through the oldest age groups,^10,19,22,25^ demonstrates minimal differences between the sexes,^25^ and is characterized by higher education and socioeconomic status.^6,7,10,22^

As a contributor to successful aging models, PIL is highly correlated with resilience and social support—perhaps tapping into some aspect of inner strength.^2^ Resilience (ie, the ability to adapt to changes and cope with life's challenges) has been recognized as a concept central to successful aging in promoting recovery from negative stressors, reducing depression, and aiding in the reestablishment of positive life balance and improved perceived health over time.^1–5^ In addition, ongoing social support and quality relationships constitute a consistent element of optimal human functioning and psychological well-being.^3–5,22^ Social support, actual or perceived, serves to buffer stress, provides aid in coping with life's changes, and augments self-perceptions of value of an individual's life.^4,5,14,16,25^

PIL may function as a positive marker for high engagement in life and may be a measure of intrinsic motivation to take care of one's health, either for its own sake or as a strategy to achieve higher life goals.^2,21,24^ Those with higher PIL appear to share a commitment to positive health behaviors (eg, physical activity^11,21,24^) and consequently benefit from consistently better health. Those with higher PIL self-report better perceptions of their personal health,^22^ higher utilization of recommended preventive services,^20^ and fewer nights spent in a hospital.^20^ To date, no studies examining PIL have utilized administrative claims-based outcomes (eg, preventive services compliance, health care utilization, health care expenditures).

Objective measures of health status derived from administrative health care claims provide a biomedical aspect of aging well based on interactions with medical services and associated expenditures: an assessment of service types, diagnosis of chronic conditions, and the severity of those conditions.^1^ Utilization of recommended preventive services can be tracked along with adherence with medication protocols, providing an indication of the individual's attention to self-care.^14,21,24^ Meanwhile, subjective reports of quality of life (QOL) include self-rated aspects of physical and mental health including physical limitations and the impact of pain or mental health on accomplishments of daily activities.^1,2^ QOL measures may provide the better assessment, however, of an individual's overall perceived health, as well as success in adjusting over time to the many health, social, and psychological issues that present with aging.

Despite the benefits of increasing PIL, successful interventions have yet to be developed. Researchers suggest that appropriate interventions could be based on common characteristics of those with higher PIL. For example, because PIL is associated with engagement in goal-driven, meaningful activities,^6,7,10,20–22,24^ encouraging these activities may help those with lower PIL. Other approaches focused on meaning in life have shown limited success but lack scalability. These include life reviews^26^; cognitive behavioral therapies,^22^ problem-solving, and or coping strategies designed to reduce stress^4^; and meaning-centered psychotherapy.^27^

PIL is a relatively new research focus that attempts to understand the drivers of optimal health throughout older adults' lifetimes. No published research studies to date were found investigating PIL among older adults with Medicare Supplement plans (ie, Medigap).^28^ Although most (about 90%) of those with original fee-for-service Medicare coverage have some type of supplemental insurance coverage, about 28% (currently about 10.2 million adults) have purchased Medigap coverage.^28^ Because this population may differ from general older adult and/or specifically Medicare populations, it was of interest to determine the prevalence of PIL, associated characteristics, and selected health outcomes linked with designated levels of PIL. This study adds to the PIL literature in considering the prevalence of defined low, medium, and high levels of PIL in a nationally representative Medigap population utilizing both subjective and objective health outcomes.

Thus, the primary objective was to estimate the prevalence and associated characteristics of low, medium, and high levels of PIL among AARP Medicare Supplement insureds. The secondary objective was to measure the impact of PIL levels on selected health outcomes: health care utilization and expenditures, preventive services compliance, and QOL. This research was covered under New England IRB (NEIRB) number 120160532.

## Methods

### Sample selection

In 2015, approximately 4 million Medicare insureds were covered by an AARP Medicare Supplement plan insured by UnitedHealthcare Insurance Company (for New York residents, UnitedHealthcare Insurance Company of New York). These plans are offered in all 50 states, Washington, DC, and various US territories. From August through September 2016, AARP Medicare Supplement insureds were mailed surveys using a nationally randomized stratified methodology. To be eligible for this study, insureds must have been in a plan for a minimum of 12 months and must have been at least 65 years of age prior to completing the survey. The random sample included 16,000 insureds, oversampling sicker populations. Of survey respondents (N = 4664), those who did not match with eligibility files (N = 3) and those who did not answer the PIL questions (N = 98) were excluded. Thus, the final study population included 4563 survey respondents. Their responses were then weighted to adjust for nonresponse bias and to be nationally representative. This weighted study sample will be the focus of the following analyses.

### Survey

The mailed survey (49 questions) was developed by UnitedHealthcare and AARP Services, Inc. to assess psychosocial aspects of health including PIL, resilience, social support, health literacy, reliance on faith, as well as their impact on QOL (mental and physical components). Other questions included financial stress (ie, worried about paying bills) and living alone. The survey was mailed to the stratified sampling in August 2016 with a repeat mailing in September 2016 to those who had not yet responded.

### Purpose in life

PIL was measured using 7 items adapted from the National Institutes of Health Tuberculosis Meaning and Purpose Scale Age 18+.^29^ Responses, ranging from 1 to 5, were scored if at least 4 of the 7 questions were completed, and were averaged across the questions answered to give a range of scores from 1 to 5. Three levels of PIL were categorized as follows: low (scores of 1 to <3.5); medium (scores of 3.5 to <4), and high (≥4). High PIL was defined as answering *agree* or *strongly agree* on each of the 7 questions; low and medium subgroups were defined based on the remaining score distribution using a cut point at the 50^th^ percentile.

### Resilience

Resilience was measured using the 6-item Brief Resilience Scale.^30^ Responses, ranging from 1 to 5, were scored if at least 3 of the 6 questions were completed, and were averaged across the questions answered to give a range of scores from 1 to 5. Resilience was then dichotomized as follows: low (scores 1 to <4) and high (scores ≥4). High resilience was defined as answering *agree* or *strongly agree* to each of the 6 questions; low was defined as the remaining subgroup.

### Social support

Social support was measured using the 12-item Interpersonal Support Evaluation List.^31^ Responses, ranging from 1 to 4, were scored if at least 6 of the 12 questions were completed, and were averaged across the questions answered to give a range of scores from 1 to 4. Social support was dichotomized at the median^31^ as: low (scores of 1 to <3.5) and high (scores ≥3.5).

### Health literacy and reliance on faith

Health literacy was measured with the single validated question asking for confidence level in filling out medical forms.^32^ Responses of *extremely* and *quite a bit* were utilized to define high health literacy. Reliance on faith was measured with a single question: *I rely on faith when times get tough*. Responses of *strongly agree* and *very strongly agree* were utilized to define high reliance on faith.

### Covariates

Covariates were included to describe individuals with low, medium, and high PIL. These covariates included measures of demographics, socioeconomic factors, health status, and other characteristics taken from health plan eligibility and administrative medical claims.

Demographic questions included age and sex. Age groups were defined as: 64–69, 70–79, ≥80 years. Geographic regions (Northeast, South, Midwest, and West), urban location (urban and other), and low, medium, and high minority areas were geocoded from zip codes. AARP Medicare Supplement plan types were grouped by cost-sharing levels, including high-level coverage plans with minimal co-payments or deductibles, medium-level coverage, and all other plans. Four categories of health status were defined based on Hierarchical Condition Category (HCC) scores.^33^ This score is used by Centers for Medicare & Medicaid Services to risk adjust medical payments across various medical plans according to the health status of the different insured populations and can be used as a surrogate measure of the health status of selected subgroups in Medicare populations. HCC subgroups were defined as follows and utilized to control for health status: HCC scores <0.5; HCC scores 0.5 to <1.2; HCC scores 1.2 to <2.8; and HCC scores ≥2.8. Living alone was assessed from the question, *How many people live with you?* A response of *0* was used to define living alone.

### Health care utilization and expenditures

Health care utilization was defined from administrative medical claims as an inpatient admission (IP) or emergency room (ER) visit within the 1 year pre survey. Health care expenditures were defined as paid claims from the same time period aggregated from Medicare, Medicare Supplement, and patient out-of-pocket paid amounts. Prescription drug expenditures included AARP Medicare drug paid claims and patient co-payments for those also enrolled in an AARP Medicare Part D Rx plan (about 55% of the overall sample).

### Quality of care measures: care pattern and medication compliance

Individual-level care pattern and medication utilization were used as quality of care measures determined from evidence-based recommendations of care for chronic conditions. Care patterns included annual physician visits for those with chronic conditions, along with recommendations for preventive care (eg, regular monitoring of biometric values with lab tests, diabetic vision and foot examinations). Survey respondents were linked to Evidence-Based Medicine (Symmetry EBM Connect Version 8.3; Optum, Eden Prairie, MN) software. This software program was developed to calculate utilization of care patterns from health care claims and medications from pharmaceutical claims using a defined set of rules for evidence-based care associated with various chronic conditions. Ten common chronic conditions (asthma, coronary artery disease, chronic obstructive pulmonary disease, congestive heart disease, depression, diabetes, hypertension, hyperlipidemia, osteoporosis, and rheumatoid arthritis) were included in this analysis. To be considered “compliant,” individuals must have utilized recommended care patterns by chronic condition category or have been adherent with recommended medication protocols assessed for 1 year prior to survey completion. The number of care patterns or medications for which each individual was noncompliant across all categories of his or her chronic conditions (eg, heart disease, diabetes, depression) were counted.

### Veteran's RAND 12-item QOL scale

The 12-item Veteran's RAND health status/QOL scale, which has been validated for use in older populations, was used to examine the impact of PIL on QOL.^34^ Physical and mental health component scores (PCS and MCS) were calculated. For these scales, a score was calculated if at least 50% of the items in the scale were completed. QOL scale scores ranged from 0 (worst possible) to 100 (best possible). To compare with the general US population or other Medicare populations, the scores were transformed to “standardized scores” with a mean of 50 and a standard deviation of 10.^35^

### Statistical models

#### Weighting to adjust for survey nonresponse bias and stratified sampling

Propensity weighting was used to adjust for potential selection bias often associated with survey responses to enhance the generalizability of these findings. The propensity weighting utilized available information about the aforementioned demographic, socioeconomic, and health status variables that could potentially influence survey response. This information was used to estimate the underlying probability of survey response for each individual. That estimated probability was then used to create and apply a weighting variable to the data to make those who did respond better resemble all eligible insureds who received the survey. The utility of such propensity weighting models to adjust for external validity threats is described elsewhere.^36,37^ In addition, the survey responses were weighted to achieve national representation of the entire AARP Medicare Supplement population with 1 year of plan eligibility.

Characteristics associated with PIL levels were determined using multivariate logistic regression models for medium and high versus low levels of PIL weighted to adjust for survey nonresponse and stratified sampling. The psychosocial variables (ie, resilience, social support, reliance on faith) and health literacy were highly correlated and, consequently, were entered into separate regression models adjusting for other covariates. Covariates included all of those variables listed in Table 1.

**Table 1.** Unadjusted Demographics for Weighted Study Population by Purpose in Life Levels

		*PIL*			
	*All mean or %*	*Low mean or %*	*Medium mean or %*	*High mean or %*	P
Number	15,680	3793	3306	8581	
Demographic variables					
Sex					
Male	41.1	40.8	40.8	41.4	0.79
Female	58.9	59.2	59.2	58.7	
Age	76.5	78.3	77.2	75.6	<0.0001
65–69	21.5	17.2	19.9	24.0	<0.0001
70–79	45.8	40.2	43.6	49.1	
≥80	32.7	42.6	36.5	26.9	
Minority (from zip code)					
Low	52.5	52.2	55.6	51.4	0.001
Median	44.4	44.8	41.8	45.2	
High	3.1	3.0	2.6	3.4	
Location (from zip code)					
Urban	82.0	80.4	82.2	82.7	0.007
Other	18.0	19.6	17.9	17.3	
Region (from zip code)					
Midwest	16.8	18.0	18.1	15.7	<0.0001
Northeast	23.5	24.0	23.6	23.3	
South	39.3	38.9	36.9	40.5	
West	20.4	19.1	21.3	20.6	
Plan type					
High coverage	72.0	71.5	71.4	72.4	0.53
Midrange coverage	3.6	3.8	3.4	3.7	
Other	24.4	24.7	25.2	24.0	
Claims-based variables					
Health status pre survey					
HCC <0.50	25.7	16.7	22.6	30.9	<0.0001
HCC 0.50 to <1.20	42.3	38.5	43.6	43.5	
HCC 1.20 to <2.80	24.9	33.7	26.2	20.6	
HCC ≥2.8	7.0	11.1	7.7	5.0	
Inpatient admission (annual)	13.5	17.5	13.5	11.8	<0.0001
Emergency room visits (annual)	29.7	34.3	30.5	27.4	<0.0001
Medical expenditures—pmpm	$998	$1280	$1025	$862	<0.0001
Number with Part D coverage	8555	2104	1815	4637	
Drug expenditures—pmpm	$220	$270	$248	$187	0.01
Survey variables					
Purpose in life score	3.9	3.0	3.7	4.4	<0.0001
Resilience score	3.8	3.3	3.7	4.1	<0.0001
Low (score <4)	49.8	76.8	59.4	34.	<0.0001
High (score ≥4)	48.6	20.7	39.0	64.7	
Social support score	3.4	2.9	3.3	3.6	<0.0001
Low (score <3.5)	48.2	78.8	59.5	30.3	<0.0001
High (score ≥3.5)	50.1	18.4	38.6	68.5	
Reliance on faith					
High	59.9	41.9	58.3	68.6	<0.0001
Low	40.0	58.2	41.7	31.4	
Financial stress					
High	13.5	23.2	13.7	9.2	<0.0001
Low	86.5	76.8	86.3	90.8	
Health literacy					
High	74.8	58.9	74.1	82.1	<0.0001
Inadequate	25.2	41.1	25.9	17.9	
Live alone					
Live alone	29.0	35.5	31.3%	25.3%	<0.0001
Live with others	71.0	64.5	68.7%	74.7%	
QOL (VR-12)					
PCS	44.0	38.4	43.1	46.8	<0.0001
MCS	54.6	47.0	54.0	58.1	<0.0001

HCC, Hierarchical Condition Category; MCS, mental component score; PCS, physical component score; PIL, purpose in life; pmpm, per member per month; QOL, quality-of-life; VR-12, Veterans RAND 12-Item QOL scale.

Health care utilization (inpatient admissions and emergency room visits), health care expenditures, preventive services compliance, and QOL for low, medium, and high PIL levels were determined, weighted, and regression adjusted for demographic, socioeconomic, and survey response variables listed in Table 1.

## Results

Overall, 4664 AARP Medicare Supplement insureds responded to the survey (29.2% response rate). Of these, 97.8% (N = 4563) met the eligibility criteria and were included in the study. Responses were subsequently weighted to a nationally representative population of 15,680. Weighted survey respondents were mostly female, 70–79 years of age, and more than half were white (52.5%). The prevalence of the HCC health status groups (HCC scores <0.5; HCC scores 0.5 to <1.2; HCC scores 1.2 to <2.8; and HCC scores ≥2.8) were as follows: 25.7%, 42.3%, 24.9%, and 7.0%, respectively. Among survey respondents, the prevalence of low, medium, and high PIL levels was 24.2%, 21.1% and 54.7%, respectively (Table 1).

### Characteristics associated with PIL levels: medium and high vs. low

The results of the multivariate logistic models predicting PIL levels are shown in Table 2. The strongest characteristics of medium and high PIL from the separate regression models were high social support followed by high resilience, strong reliance on faith, and high health literacy. Among the other significant covariates, good health as indicated by HCC scores <0.5 and HCC scores 0.5 to <1.2 was also a strong characteristic of medium and high PIL. Financial stress, living alone, and ages ≥80 years (high only) were significantly associated with reduced medium and high PIL: 30% to 50% reduced likelihood to have medium PIL and 10% to 70% less likely to have high PIL.

**Table 2.** Characteristics Associated with Medium and PIL Compared to Low PIL

	*Medium purpose in life*		*High purpose in life*	
*Separate regression models*^a^	*Odds ratio*	P	*Odds ratio*	P
Social support—high	2.59	<0.0001	8.45	<0.0001
Resilience—high	2.23	<0.0001	5.98	<0.0001
Reliance on faith—high	2.24	<0.0001	3.73	<0.0001
Health literacy—high	1.79	<0.0001	2.55	<0.0001

*Covariates*^b^	*Odds ratio ranges*	P	*Odds ratio ranges*	P
HCC score <0.5	1.62–1.87	<0.001	2.66–3.54	<0.001
HCC score 0.5 to <1.2	1.44–1.59	<0.001	1.95–2.35	<0.001
HCC score 1.2 to <2.8		NS	1.28–1.38	<0.001
Live alone	0.87–0.89	<0.01	0.69–0.89	<0.001
Age ≥80 years		NS	0.70–0.81	<0.001
Financial stress	0.50–0.61	<0.001	0.30–0.47	<0.001

^a^ Controlled for age, sex, minority status, urban location, geographic region, health plan type, live alone, HCC category, and financial stress.

^b^ Covariate odds ratio ranges from 4 psychosocial models listed. Only significant covariates are shown.

HCC, hierarchical condition category; NS, not significant; PIL, purpose in life.

### Association of PIL levels with health care utilization and expenditures

There was a dose-response relationship for health care utilization for inpatient admissions and emergency room visits (all medium and high versus low PIL level comparisons significant) across low, medium, and high PIL levels (Table 3). A similar dose-response relationship was evident for paid medical and drug expenditures (all medium and high vs. low PIL level comparisons significant with the exception of medium PIL drug expenditures compared to low): as PIL levels increased, medical and drug expenditures significantly decreased (Table 3). High levels of PIL demonstrated the largest association with utilization and expenditures compared to low levels (IP −4.3 percentage points (pp); ER −4.1 pp; medical expenditures -$418 per member per month (pmpm); prescription drug expenditures -$75 pmpm). Medium levels of PIL were associated with significantly lower utilization and expenditures compared to low PIL but were not as impactful as high PIL levels (IP −3.3 pp; ER −2.5 pp; medical expenditures -$245 pmpm; drug expenditures not significant).

**Table 3.** Health Outcomes Associated with Purpose in Life Levels

	*PIL*			P	
*Regression-adjusted estimates*	*Low*	*Medium*	*High*	*Medium vs. low*	*High vs. low*
Health care utilization					
Inpatient admissions (annual)	16.8%	13.5%	12.5%	<0.0001	<0.0001
Emergency room visits (annual)	32.8%	30.3%	28.7%	0.02	<0.0001
Health care expenditures					
Medical (pmpm)	$1288	$1043	$870	<0.0001	<0.0001
Drug (pmpm)	$269	$250	$194	0.33	<0.0001
Preventive services					
Care pattern (% compliant)	19.6	21.1	23.3	0.14	<0.0001
Medication adherence (% compliant)	64.5	70.2	71.6	0.003	<0.0001
QOL (VR-12)					
PCS	39.0	43.1	46.0	<0.0001	<0.0001
MCS	47.4	54.0	57.8	<0.0001	<0.0001

MCS, mental component score; PCS, physical component score; PIL, purpose in life; pmpm, per member per month; QOL, quality of life; VR-12, Veterans RAND 12-Item QOL scale.

### Association of PIL levels with preventive services compliance

Preventive service care patterns for those with high PIL were significantly higher compared to those with low PIL (+3.7 pp) (Table 3). There were no significant improvements in care pattern utilization associated with medium PIL. Meanwhile, adherence with medication protocols was significantly higher for those with medium and high PIL (+5.7 pp and +7.1 pp, respectively).

### Association of PIL levels with QOL (PCS and MCS)

There was a strong dose-response relationship for increasing PCS and MCS across low, medium, and high levels of PIL: as PIL increased, PCS and MCS also increased (PCS: +4.1 and +7.1; MCS: +6.6 and +10.4 for medium and high PIL compared to low PIL, respectively) (Table 3).

## Discussion

In this population of AARP Medicare Supplement insureds, 24.2%, 21.1%, and 54.7% were assessed as having low, medium, or high PIL, respectively. Previously, most researchers have utilized the PIL score as a continuous variable^6–10,14,15,17,19,20^ or categorized scores based on a given distribution (eg, tertiles).^20^ No other studies categorized to designated PIL levels, and thus a direct comparison of the prevalence of PIL levels to other studies was not possible. However, the average PIL score of 3.9 in this study was generally in the same range as other studies with 5 responses.^6,7,19^ As in other studies, there were minimal demographic differences with the exception of those 80 years of age and older having lower PIL.^6,7,17,23^ Overall, those with high PIL were more likely to have high social support, high resilience, strong reliance on faith,^10,38^ high health literacy, and better health. Those with lower PIL were more likely to live alone^10,22^ and to report high financial stress (worry about paying bills).^38^

The strongest characteristics associated with medium and high PIL were the psychological constructs of resilience and social support. The strong correlations between PIL, resilience, and social support have been documented in other studies as well.^2,7,8,19^ The present study utilized separate regression models to avoid underreporting the impact of these characteristics and their associations with PIL.^7,8,19^ As presented in this study, PIL provides the overarching psychological construct, supported by resilience, social support, reliance on faith, and health literacy, which subsequently is associated with positive health outcomes. Resilience appears to be positioned as essential to successful aging in dealing with negative stressors.^1–4^ Like PIL, resilience scores (across different measures) appear to be stable over time.^39^ When resilience does change, however, those changes have been associated with directional changes in fatigue, sleep disturbances, physical functionality, and depression.^39^ Likewise, social support and maintaining quality relationships over time appear to be essential elements critical to positive psychological well-being and successful aging.^14–16^

Financial stress and living alone, as measures of chronic stress, demonstrated powerful negative impacts on PIL and were significantly associated with lower PIL. The PIL stress buffering mechanism suggested in other studies^14,15,40^ appears to be associated with a quicker recovery from negative stressors, thus avoiding longer term negative psychological states.^40^ Considering these results, PIL may be more effective for buffering acute and/or shorter term stressors rather than chronic stressors.^14,15^ This interpretation would be consistent with a study indicating that religious involvement, but not meaning in life, buffered financial strain.^38^ A more thorough examination of the impact of different types of stress on PIL and effective coping mechanisms may be warranted.

A dose-response relationship of decreasing inpatient admissions, emergency room visits and health care expenditures was evident across PIL levels. Medium and high PIL were associated with fewer inpatient admissions and emergency room visits, lower medical expenditures, and lower drug expenditures. No other studies were found that utilized administrative claims data to assess health status associated with PIL. One study reported 23% and 33% lower self-reported nights in the hospital associated with the second and top tertiles of PIL scores, respectively, but no attempt was made to monetize these differences.^20^ The self-reported nights in the hospital reductions associated with the middle and top tertiles were in agreement with the measured inpatient admissions utilization in the present study: reductions of 20% and 26% for medium and high PIL categories, respectively.^20^ Lower health care utilization and expenditures would be consistent with a positive biomedical view of successful aging.

Preventive services utilization was measured separately as care patterns and as adherence with medication protocols. Those with high PIL were more likely to be compliant with care patterns associated with their chronic conditions (19% increased); medium PIL showed no significant relationship with care patterns. Furthermore, those with medium and high PIL had significantly higher adherence rates with medication protocols: 9% and 11% higher, respectively. These results were similar to those in a study utilizing self-reported utilization of recommended preventive services such as mammograms, colonoscopies, and cholesterol tests. This study reported a dose-response across tertiles of PIL scores with significantly increased utilization for those in the highest tertile but not generally associated with significance for the second tertile.^20^ Although no other studies were found directly assessing PIL and health literacy, the association with high health literacy could promote better health decision making and reflect more effective management of existing chronic conditions.

Finally, the assessment of QOL provides a subjective perspective of the individual's perception of his or her physical and mental health. Those with medium and high PIL reported higher PCS and MCS: 11% and 18% higher PCS and 14% and 22% higher MCS, respectively. These results suggest that PIL improves both physical and mental health. In a similar study that assessed PIL and resilience, along with other psychological indicators, PIL likewise impacted both PCS and MCS; resilience, however, only significantly impacted MCS.^2^ Of note, in a 7-year longitudinal study, higher QOL was predicted by psychological resources including a sense of purpose, self-efficacy, and optimism, but not by biomedical or social approaches to healthy aging.^1^

Interventions focused exclusively on PIL have yet to be developed, although several researchers have recommended a focus on goal-driven, meaningful activities defined as hobbies, volunteerism, religious involvement, or physical activities.^6,7,10,20–22,24^ As interventions are tested, however, perhaps the target population of those with high PIL would be worthy of suitable programs to help maintain higher levels of PIL. To date, while theoretically improving PIL should be possible, the PIL scores are very stable so expectations would predict relatively small changes with any intervention.^10,15,19,25^ Alternative approaches could focus on stress reduction and coping or problem-solving strategies as another methodology to indirectly improve PIL.^4^

This study has some limitations. This population of AARP Medicare Supplement insureds may not generalize to all older adults or other Medicare Supplement beneficiaries. Although adjustments were made for survey nonresponse and the random stratified sampling methodology, the response rate of 29% was relatively low. This study focused on PIL as the key driver, supported by resilience, social support, reliance on faith, and health literacy, associated with positive health outcomes. These psychological variables were highly correlated; consequently, separate models were utilized for each factor to avoid underreporting the associations. However, in cross-sectional studies such as this, the directionality of associations may be debatable. Numerous prospective studies from 2 to 8 years would strongly suggest that high PIL results in better health and less disability.^6–11,19^ However, the argument could be made that higher PIL is a result of better health and physical functionality rather than better health and reduced disability as the outcome. Strengths of this study include the examination of both subjective and objective measures of health outcomes with the consistency of the results adding credibility to these conclusions.

## Conclusions

Overall, 24.2%, 21.1%, and 54.7% of AARP Medicare Supplement insureds reported low, medium, and high PIL, respectively. The strongest characteristics associated with medium and high PIL were social support, resilience, reliance on faith, health literacy, and better health. Those with medium and high PIL had significantly lower health care utilization and expenditures, increased compliance with preventive services, and higher QOL. Regardless of the directionality, PIL is strongly associated with better physical and mental health outcomes among older adults. Thus, interventions to improve and, perhaps more appropriately, maintain higher levels of PIL over time may be warranted as a key contributor to successful aging.
